# Self-Reported vs Objective Cerebrovascular Burden: Association with Depression and Cognitive Performance

**DOI:** 10.1093/geroni/igaf122.3057

**Published:** 2025-12-31

**Authors:** Emma Churchill, Daniel Paulson, Nichole Lighthall, Apollonia Lysandrou, Nicole McClure, Madison Maynard, Quinn Allen, Nick James

**Affiliations:** University of Central Florida, Orlando, Florida, United States; University of Central Florida, Orlando, Florida, United States; University of Central Florida, Orlando, Florida, United States; University of Central Florida, Orlando, Florida, United States; University of Central Florida, Orlando, Florida, United States; University of Central Florida, Orlando, Florida, United States; University of Central Florida, Orlando, Florida, United States; VA Orlando Health Care, Orlando, Florida, United States

## Abstract

**Introduction:**

Cerebrovascular burden (CVB) contributes to neurocognitive and behavioral decline in aging. While MRI is the gold standard for assessing CVB, its availability in large community samples is limited. Previous studies have used biomarkers (e.g., A1C) or self-reported comorbidities (e.g., diabetes) to assess CVB. This study evaluates the concordance of these methods, hypothesizing that self-reports serve as a valid alternative to biomarkers in predicting depressive symptoms and cognitive performance.

**Methods:**

Data from wave 16 of the Health and Retirement Study (HRS), a nationally representative sample of older adults, were analyzed. Biomarker (A1C, cholesterol, blood pressure, C-reactive protein, cystatin C) and self-reported CVB variables (heart conditions, hypertension, hypercholesterolemia, smoking) were collected. Depression and cognition were assessed using the CES-D 8 and the Harmonized Cognitive Assessment Protocol (HCAP), respectively. Multiple stepwise regression analyses examined relationships among self-reported and objective CVB, depressive symptoms, and cognitive functioning.

**Results:**

The sample included 916 (60.2% female) participants with an average age of 75.02 (SD = 7.18). Self-reported CVB predicted depressive symptoms and cognitive performance for Symbol Digit Modalities, Raven’s Matrices, and Trail Making Test-B (p < .05). Objective CVB also significantly predicted cognitive performance.

**Discussion:**

Self-reported CVB may offer comparable (or greater) predictive value than biomarkers alone, especially for measures of mood. These findings support the use of self-reported cerebrovascular morbidities for the study of cognitive aging as they could reduce costs and enhance the utility of large scale studies. This highlights the value of integrating both methods when feasible.

